# A realist inspired barriers and facilitators evaluation of the co-creation process in the Youth-centred Participatory Action Project

**DOI:** 10.1093/heapro/daag134

**Published:** 2026-09-10

**Authors:** Maria Fernanda S Souza, Mai Chinapaw, Catarina Santos Silva, Leto Demetriadou, Line Madsen, Vongani Maluleke, Lethukuthula Ndwandwe, Anita Okafor, Adefunke Ogunleye, Jesse Vargas, Giuliana Longworth, Teatske Altenburg, Leonie Klaufus, Dayo Omotoso, Adewale Oyeyemi, Lisa Ware, Antonio Palmeira, Marlene N Silva

**Affiliations:** Research Center in Sports, Physical Education, Exercise and Health (CIDEFES), Universidade Lusófona, Campo Grande, 376, Lisboa 1749-024, Portugal; CIFI2D, Faculdade de Desporto, Universidade do Porto, Rua Dr. Plácido Costa 91, Porto 4200-450, Portugal; Public and Occupational Health, Amsterdam UMC Location VUmc, De Boelelaan 1117, 1081 HV Amsterdam, The Netherlands; Research Center in Sports, Physical Education, Exercise and Health (CIDEFES), Universidade Lusófona, Campo Grande, 376, Lisboa 1749-024, Portugal; CIFI2D, Faculdade de Desporto, Universidade do Porto, Rua Dr. Plácido Costa 91, Porto 4200-450, Portugal; Public and Occupational Health, Amsterdam UMC Location VUmc, De Boelelaan 1117, 1081 HV Amsterdam, The Netherlands; Department of Sports Science and Biomechanics, Faculty of Health Sciences, Southern Denmark University, Campusvej, 55, Odense M DK52-30, Denmark; Department of Paediatrics, School of Clinical Medicine, University of the Witwatersrand, Wits Medical School, 7 York Road, Parktown, Johannesburg 2193, South Africa; Department of Paediatrics, School of Clinical Medicine, University of the Witwatersrand, Wits Medical School, 7 York Road, Parktown, Johannesburg 2193, South Africa; Department of Physiotherapy, Redeemer's University, off Gbongan - Oshogbo Road, Ede, Osun State 232101, Nigeria; Gender and Education Department, Value Re-Orientation for Community Enhancement (VARCE), 25, Bola Famoyin Lane, Dada State, Osogbo, Osun State 230261, Nigeria; Public and Occupational Health, Amsterdam UMC Location VUmc, De Boelelaan 1117, 1081 HV Amsterdam, The Netherlands; School of Health and Life Sciences, Glasgow Caledonian University, Glasgow, Cowcaddens Road, Glasgow G4 0BA, United Kingdom; Public and Occupational Health, Amsterdam UMC Location VUmc, De Boelelaan 1117, 1081 HV Amsterdam, The Netherlands; Public and Occupational Health, Amsterdam UMC Location VUmc, De Boelelaan 1117, 1081 HV Amsterdam, The Netherlands; Department of Anatomy, Redeemer's University, off Gbongan - Oshogbo Road, Ede, Osun State 232101, Nigeria; College of Health Solutions, Arizona State University, 550 N, 3rd Street, Health North Building, Suite 501, Phoenix, AZ 85004, United States; Department of Paediatrics, School of Clinical Medicine, University of the Witwatersrand, Wits Medical School, 7 York Road, Parktown, Johannesburg 2193, South Africa; Research Center in Sports, Physical Education, Exercise and Health (CIDEFES), Universidade Lusófona, Campo Grande, 376, Lisboa 1749-024, Portugal; CIFI2D, Faculdade de Desporto, Universidade do Porto, Rua Dr. Plácido Costa 91, Porto 4200-450, Portugal; Research Center in Sports, Physical Education, Exercise and Health (CIDEFES), Universidade Lusófona, Campo Grande, 376, Lisboa 1749-024, Portugal; CIFI2D, Faculdade de Desporto, Universidade do Porto, Rua Dr. Plácido Costa 91, Porto 4200-450, Portugal

**Keywords:** co-creation, participatory research, adolescents, movement behaviours, physical activity, health inequities, realist thinking

## Abstract

This study examined barriers and facilitators of the first phase of the co-creation process as part of the Youth-centred Participatory Action (YoPA) project, a multi-country co-creation initiative to address health inequities by engaging adolescents in Denmark, Nigeria, South Africa, and the Netherlands. Using a qualitative design informed by implementation science and realist thinking, we analysed data from 22 transcripts of semi-structured interviews with researchers, facilitators, and external stakeholders, alongside 61 logbooks from co-creation sessions. Data were coded deductively using the PROSECO process-evaluation framework, followed by the development of exploratory Context–Mechanism–Outcome configurations. Findings highlight four predominant areas shaping the co-creation process: group dynamics, partnerships, contextual influences, and resources and support. Facilitators included psychologically safe spaces, horizontal communication, consistent facilitation, strong institutional partnerships, motivation driven by purpose and belonging, and access to organizational resources and staff support. Barriers were associated with socioeconomic adversity, unstable infrastructures, logistical constraints, competing priorities, uneven engagement, and challenges in translating complex systems-thinking concepts into youth-friendly practice. Realist interpretations suggest that when structured participation, trust, and relational support were present, they activated mechanisms of ownership, agency, and sustained engagement; conversely, low-resource contexts and activities overload hindered participation, feasibility, and engagement. This study contributes empirical evidence on co-creation processes with adolescents across diverse settings. It demonstrates the combined value of the PROSECO framework and realist-inspired analysis for evaluation and understanding of how context and mechanisms shape co-creation in health-related research. Implications include the need for resourcing relational work, and designing context-sensitive strategies when conducting co-creation with adolescents.

Contribution to Health Promotion

- This study offers useful lessons for future co-creation research in non-communicable diseases (NCD) prevention, physical activity, health, and wellbeing, focused in adolescents.
- It tests a novel process evaluation framework and shows how it can support co-creation in health-related initiatives.
- It identifies, in the co-creation process with adolescents, barriers that can be planned for early and facilitators that can be strengthened from the start.
- It links practical findings with theory, helping explain how co-creation works, in multiple contextual settings, which can strengthen the uptake of this participatory method in health programmes.

## Introduction

Adolescent physical inactivity is a growing global concern. Over 80% of young people aged 11–17 do not meet the World Health Organization (WHO) recommendation of 60 min of moderate intensity physical activity per day, and one in five adolescents are classified as overweight (WHO 2022, World Health Organization Regional Office for Europe 2022). Adolescent mental health is another global issue, as one in seven 10- to 19-year-olds experience mental conditions (WHO 2025). With 1.3 billion adolescents worldwide (UNICEF 2019), this life stage, marked by rapid biological and social transitions, is increasingly recognized as foundational for lifelong health (Sawyer *et al*. 2012).

Despite an increasing number of interventions aimed at promoting youth physical activity and mental health (Biddle *et al*. 2019, Rodriguez-Ayllon *et al*. 2019, Hale *et al*. 2021), the problem of physical inactivity persists, with large disparities across subgroups. Adolescents experience growing autonomy, strong peer influence, and diverse social and environmental conditions, which may challenge the effectiveness of traditional, top-down, adults customized health interventions (e.g. Frank 2006, Gibbs *et al*. 2010). Participatory approaches, including co-approaches, are therefore highly relevant for public health efforts (Baum *et al*. 2006, Verloigne *et al*. 2023) and for sports, exercise, and health sciences (Smith *et al*. 2023).

Defined by Vargas *et al.* (2022) as ‘the collaborative approach of creative problem solving between diverse stakeholders at all stages of an initiative, from the problem identification and solution generation through to implementation and evaluation’, co-creation has the potential to generate research results that are more applicable, context-sensitive, and implementable (Greenhalgh *et al*. 2016). Agnello *et al*. (2025) identified several benefits (and challenges) of applying co-creation methods in public health, and recent systematic reviews and meta-analyses (Halvorsrud *et al*. 2021, Anieto *et al*. 2025) demonstrated the potential of co-creation for the effectiveness of health interventions, especially for community identified outcomes. However, the success of co-creation depends heavily on process elements. Additional benefits of co-creation specifically for children and youth, are cognitive-emotional benefits, and acquiring leadership, agency, and interpersonal skills (e.g. Shamrova and Cummings 2017, Anyon *et al*. 2018). Madsen *et al*. (2026) found that not only participation in co-creation promotes these outcomes but also *how* the co-creation process is experienced.

Therefore, understanding what influences co-creation and its specificities is essential for advancing evidence in applying co-creation processes. Longworth *et al*. (2024a), for example, examined the barriers and facilitators for implementing co-creation in low-and-middle income countries, revealing outer and inner setting elements that are important for the success of the co-creation. Verloigne *et al*. (2025) evaluated the perspectives of facilitators (sessions’ moderators) of the co-creation process and suggested strategies to mitigate critical co-creation events that influence it. However, such process evaluations remain scarce, highlighting the need for stronger evidence based on thorough process evaluations tailored to participatory approaches (Longworth *et al*. 2024b).

The Youth-centred Participatory Action (YoPA) project (Chinapaw *et al*. 2024) provides an opportunity to examine the practice of co-creation. In YoPA, the process of co-creating social and environmental physical activity and wellbeing interventions with youth across Europe and Africa involves a stepwise approach. In phase one, the focus was in establishing and managing partnerships, building recruitment capacity, preparing and delivering co-creation sessions, and engaging stakeholders.

To support such process evaluation of the co-creation process, the PROcesS Evaluation framework for CO-creation (PROSECO) was designed (Longworth *et al*. 2025). Its comprehensive yet flexible structure supports the assessment of key aspects of the co-creation process, including group dynamics, empowerment, capacity building, facilitation, and contextual influences. Given its novelty, studies are only beginning to apply this framework, presenting an opportunity for further testing and development.

Co-creation processes can be understood as non-linear chains of causation, requiring a deeper exploration of underlying mechanisms. In realist thinking, mechanisms are understood as the hidden processes that explain how an intervention works. They represent the changes in reasoning and response that occur when particular resources or activities are introduced into a given context (Dalkin *et al*. 2015).

Realist Evaluation (Pawson and Tilley 1997) offers an approach to explain ‘which intervention components work for whom and in what circumstances’ (Jackson and Kolla 2012, p. 340). It focuses on analysing the relationships between context (C), mechanisms (M), and outcomes (O), emphasizing how specific mechanisms are triggered under particular conditions and the context-dependent nature of effectiveness (Lemire *et al*. 2020, Bonell *et al*. 2020). Realist thinking and evaluation have been increasingly applied in public health research, including participatory physical activity programmes (e.g. Willis *et al*. 2018).

When analysing a co-creation process or its implementation, understanding the mechanisms behind outcomes that act as barriers or facilitators in different contexts can help to build co-creation interventions that target specific conditions and trigger mechanisms leading to positive change.

To support this understanding, this study aimed to explore: (i) the barriers and facilitators in implementing phase one co-creation sessions across YoPA sites, using the PROSECO framework; and (ii) the mechanisms through which key barriers and facilitators shape process outcomes across different contexts, informed by realist thinking.

## Methods

### Study approach and paradigm

This study used a qualitative design informed by implementation science and inspired by realist thinking, adopting a pragmatic qualitative approach to evaluate the co-creation process in two European and two African countries.

### Study context: the YoPA project

YoPA engages adolescents in Denmark, Nigeria, South Africa, and the Netherlands (2024–2027) in co-creating local interventions to promote physical activity and wellbeing in their communities. The systems-informed co-creation process includes mapping local systems and tailoring, implementing, and evaluating interventions aimed at improving the system (Chinapaw *et al.* 2024). This study evaluated the process of co-creation of phase one of the YoPA project, which consisted of setting up the Co-creation Groups generally formed by adolescents, facilitators, and other local stakeholders, and creating local systems maps. This process involved partnering with multiple stakeholders in each site, recruitment efforts, and establishing regular co-creation sessions guided by the protocol. Co-creation sessions took place between adolescents and local adult stakeholders, including facilitators (including researchers), teachers, youth workers, and NGO staff. Adolescents mainly contributed their lived experiences and knowledge of their own communities, while facilitators and other stakeholders supported recruitment, session planning, scientific evidence, and the practical and contextual framing of the discussions. Decision-making in the process was usually shared between adolescents and adults. For example, in South Africa, the system mapping focus shifted from physical activity to mental health and peer support, as adolescents identified these issues as the most important factors affecting them and their peers’ health. The phase one of YoPA implementation in such diverse contexts offers a strong basis for examining early barriers, facilitators, and underlying mechanisms.

### Sampling, data sources, and data collection

All consortium researchers directly involved in local implementation of YoPA co-creation sessions were invited for interviews and most of them (16 of 18) were interviewed. External stakeholders involved in the co-creation implementation (e.g. teachers, youth workers, and NGO staff) were recruited through convenience sampling, ensuring representation from each site. The semi-structured interview guide (Supplementary material S2) was initially developed by MFS, critically discussed with MNS, and reviewed by an external researcher with expertise in realist interviews. It included questions on the co-creation process itself, the implementation of the project protocol, partnership development, contextual perspectives, and the challenges, opportunities, and outcomes observed. The guide was adapted to each participant’s role in the process, while allowing flexibility for emerging topics.

The interviews explored partnerships in the YoPA project, the planning and early implementation of the co-creation process and experiences with the initial co-creation sessions from different perspectives. It also examined contextual influences, stakeholders and youth involvement, perceived outcomes, including unintended ones, and support from the international consortium.

Semi-structured interviews were conducted online between February and April, 2025, generally recorded via Zoom and lasted between 22 and 68 min. Recordings were transcribed verbatim using TurboScribe and reviewed by the first author.

Sixty-one session logbooks from phase 1 of the co-creation process were included in the analysis. These logbooks (Supplementary material S3) functioned as a monitoring tool for facilitators to report practical information (e.g. number of session participants, date, and duration), session planning and implementation (e.g. objectives achieved, activities executed as planned), as well as reflections capturing contextual factors, learnings, and insights. For this study, only the reflective parts of the logbooks, containing facilitators’ written observations, challenges, and insights were analysed. All the facilitators who wrote the logbook notes were also interviewed for this study.

### Ethical procedures

Ethical approval for this study was obtained from the Ethics Committee of the Faculty of Physical Education and Sport, Lusófona University, Portugal (F2625).

All interviewees provided written informed consent and were given opportunities to ask questions throughout data collection.

### Data analysis

Two main steps were taken for data analysis: (i) a framework-driven thematic analysis on barriers and facilitators and (ii) the exploration of Context–Mechanism–Outcome (CMO) configurations (Jagosh 2019) of key barriers and facilitators.

The PROSECO Framework (Longworth *et al*. 2025) was designed to help researchers assess both the overall co-creation process and the specific methods used within it, making it suitable for planning, monitoring, and evaluating co-creation across different phases and settings.

It comprises 37 components across five broader dimensions: (i) Participation captures the extent and quality of involvement of different actors in the process, covering the components of partnership, recruitment, retention, attendance, motivation, facilitation, group dynamics, conflict management, impartial collaboration, and impactful decision-making. (ii) Experiential concerns the subjective experiences of the process by multiple actors, including the components of acceptability, clarity, resource accessibility, expectations, perceptions, experience, satisfaction, and whether the process felt user-friendly. (iii) Context addresses the broader, external conditions shaping the co-creation process, such as readiness for change, feasibility, available resources and support, and contextual influences. (iv) Impact includes the outcomes associated with co-creation, such as capacity building, empowerment, reach, personal change, knowledge integration, maintenance and systems integration, objective alignment, and mechanisms of impact. (v) Delivery refers to whether the co-creation process and its methods were implemented as intended, including aspects such as fidelity, transparency, efficiency, adaptation, useful outputs, reliability, and impact assessment.

The framework can be used as a set of selectable evaluation criteria, allowing researchers to either choose the elements most relevant to their evaluation objectives or use the whole framework and let the data guide their analysis.

In this present study, the latter approach was adopted. All interviews were thematically analysed, using the framework’s 37 components as the coding scheme for barriers and facilitators analysis, followed by the identification of sub-themes within components.

Detailed descriptions of the components, the coding book, and the audit trail of the analysis process are provided in the Supplementary material S4.

Interview transcripts and logbooks were analysed using MAXQDA24. A thematic analysis guided by the PROSECO Framework as the coding scheme was conducted. Two researchers (MFS and CSS) independently coded 10% of the interviews to ensure coding consistency and framework application, resolving discrepancies through discussion and achieving a final intercoder agreement of 71.2% using MAXQDA’s intercoder tool. Due to the large amount of data and limited human resources, the remaining data were coded and analysed by one researcher (MFS). Informed by Braun and Clarke’s (2006) thematic analysis approach, subthemes were created for the most frequently mentioned components to illustrate how they functioned as barriers or facilitators.

All data were analysed thematically. Although frequency was not treated as a proxy for relevance in this qualitative study, the most frequently mentioned components demonstrated greater depth and variation, allowing clearer identification of sub-themes and richer interpretation. To comply with conciseness, only components observed more than 30 times (*n* > 30) within each category (facilitator or barrier) are discussed here.

Following the initial analysis, an exploratory realist-inspired synthesis was undertaken. The four most frequently mentioned components were used to build exploratory CMOs, illustrating how specific contextual conditions may have triggered mechanisms that shaped observed outcomes, which offered an additional perspective on how Context and Mechanisms interact and the Outcomes they may produce.

The realist evaluation approach generally seeks to find causal explanations by building and testing theories in different contexts. Within public health, it has mostly been conducted in health systems and healthcare settings (e.g. Marchal *et al*. 2012, Howlett *et al*. 2024). Furthermore, specialists in Realist Evaluation argue that its principles and practices have great potential to contribute to decolonizing research (Renmans *et al*. 2022), as it focuses on the local contextual factors, centralizing participants’ agency and interactions.

The combination of a process evaluation framework with realist configurations can enrich theory building for co-creation interventions, as it is ‘helpful to understand how context (individual, social, cultural, and organizational) interacts with intervention components and underpinning mechanisms to bring about desired outcomes’ (Brand *et al*. 2019).

Combining realist approaches with process evaluation frameworks has been used mostly for evaluating and theorizing healthcare interventions implementation (e.g. Rycroft-Malone *et al*. 2018, Dalkin *et al*. 2021). The work of Messiha et al. (2024), argued that meta-theoretical principles in co-creation research can enrich evidence building and demonstrated how critical realism can be applied to co-creation research. Therefore, the CMOs analysis in this study represents a step forward in exploring its combination with a novel process evaluation framework focused on co-creation. For the CMO analysis, the following steps were taken: (i) initial subthemes found in the PROSECO components were synthesized into concise statements to represent the Context element; (ii) Outcomes were drawn from the first stage of analysis of interviews and logbooks (observed outcomes); (iii) Mechanisms were theoretically inferred by MFS supported by realist literature, followed by review by MNS, JV, AO, and ADO, who are from different backgrounds and contributed with their knowledge from both literature and lived experiences. This study was informed by the positionality of the first author as a Brazilian immigrant in Europe, with a background in social communication and 10 years of experience working with youth in vulnerable life situations and participatory practices. These experiences shaped her research and sensitivity to questions of power and representation. Reflexivity and contextual interpretation were strengthened through collaboration with co-authors from diverse backgrounds, familiar with the studied contexts.

## Results

Interviews were conducted with 22 adults (14 female) across all countries, including senior researchers (*n* = 8, 6 female), facilitators (*n* = 3, 2 female), PhD students involved as main or co-facilitator (*n* = 5, 3 female), and external stakeholders (*n* = 6, 3 female), such as schools or youth clubs representatives that were involved in YoPA. Interviewees were in roughly equal representation from each country (5 or 6 per country), ensuring a range of perspectives on the co-creation sessions. Additionally, 61 available logbooks from Phase 1 co-creation sessions were included for analysis, distributed as of 10 from YoPA in Nigeria, 13 from Denmark, 13 from South Africa, and 25 from The Netherlands.

### Overview of findings

Analysis identified subthemes across four PROSECO framework dimensions: Participation, Experiential, Context, and Impact. Participation-related processes and contextual conditions were most frequently discussed by interviewees, particularly group dynamics and partnerships. Experiential elements mainly reflected implementation challenges related to translating the protocol into practice. Contextual influences and the feasibility of applying co-creation were the main Context factors, while the Impact dimension highlighted mechanisms through which youth positively experienced the process. Detailed frequency of identified themes for PROSECO components across sites can be found on Supplementary Figures S1, S2, and S3 in the Supplementary material S1.

### Findings per PROSECO dimensions

#### Participation

Participation-related components were among the most frequently identified in the analysis, particularly group dynamics, partnerships, and motivation. These elements shaped how adolescents and adult stakeholders interacted within the co-creation process and influenced the extent to which they felt able to engage meaningfully.

***Group dynamics***. Group dynamics subthemes emerged as the most influential ones, acting both as facilitators and barriers. Stable routines, shared agreements, and consistent facilitation appeared to create predictability and psychological safety within the sessions. Interviewees emphasized that structured yet flexible facilitation helped adolescents feel comfortable expressing their ideas and engaging with the activities.

‘During the lessons, there was clear mutual communication that was respectful’. As a stakeholder observed, horizontal communication and reduced power hierarchies also encouraged adolescents to take on active roles within the sessions, sometimes acting as informal co-facilitators and supporting peers. ‘We've managed to […]allow them to be themselves[…] They want to play music, we allow them. We start dancing with them, even though we can't dance[…] They were free around us’.

However, group dynamics were also vulnerable to disruption. Absences, changes in facilitators, or dominant adolescents’ voices sometimes limited engagement and created tension within groups. Differences in communication abilities and facilitators’ levels of experience further shaped how effectively discussions could be moderated.

***Partnerships***. Partnerships subthemes emerged primarily as facilitators factors of the co-creation process. Pre-existing institutional collaborations and alignment with organizational interests often supported project initiation and mobilization of stakeholders. Established networks and the credibility of host organizations appeared to facilitate access to local institutions and communities.

Trust and relationship-building between researchers, facilitators, and local actors (such as teachers or youth workers) were particularly important for creating a safe and engaging process. When YoPA staff and local partners maintained open communication and regularly reflected on the sessions among them, collaboration was smoother and implementation challenges were easier to address. A stakeholder mentioned that ‘the [YoPA] staff, they are wonderful people. They are caring. They have courtesy. They have so many attributes that are good’. However, reliance on institutional partners sometimes created delays, particularly when bureaucratic procedures slowed down decision-making or required multiple approvals. Like a senior researcher observed: ‘We do not have a relationship when there's no projects. And this is something we struggle with, because every time you have to establish your context again, sometimes people get different jobs, different positions’.

***Motivation***. Motivation was observed solely as a facilitating component and reflected the various reasons why adolescents and adult stakeholders engaged in the co-creation process. Adolescents were often motivated by personal goals, such as contributing to change in their communities, gaining research experience, or developing skills that might support future opportunities. A sense of belonging within the group also appeared to strengthen motivation. A facilitator observed that ‘Overall the kids are excited. They know that they've got a project that they created. That sense of ownership and leadership is something that they pride themselves in’.

When adolescents expressed that their ideas were valued and taken seriously, they were more likely to attend sessions consistently and engage actively. Enjoyment also played an important role. Games, creative activities, and informal incentives helped maintain engagement and contributed to a positive group atmosphere.

Table 1 shows the subthemes and illustrative quotations of the Participation dimension components.

**Table 1:** Detailed subthemes and quotations illustrating components of the dimension participation.

Participation			
Component	Category	Identified subthemes	Quotations (e.g.)
Group dynamics	Facilitator	**Routines, structure, and shared agreements**: Regular meetings, clear rules, consistent formats, and session facilitators provided stability, mutual respect, familiarity, and comfort.	‘One of the things I think has worked well is that there have been consistent adults [in the sessions]. There have been fixed frameworks every time, and some rules for the room and some community rules (…)’ (Interview -External stakeholder).
		**Openness, safety, and flexibility**: A psychologically safe environment potentially enabled open expression and adaptation to challenges. Facilitators’ flexibility to youths’ needs was important for maintaining this atmosphere.	‘If (…) they [adolescents] felt like they needed a quiet place instead, they were allowed to just leave. And I think that just the possibility of leaving made them stay, (…) ‘we don't want to sit and listen to you—It's like being in school’. So we changed the focus of the sessions as well or the methods’ (Interview—PhD student).
		**Power shifting, horizontal, and inclusive communication**: Flat hierarchies between facilitators and youth encouraged co-facilitation, peer support, confidence, and autonomy. Treating adolescents as co-researchers with ownership and influence strengthened collaboration and shared responsibility.	‘One of the participants informally took on the role of co-facilitator—offering additional explanations to participants (…) and helping manage the group’ (Logbook—facilitator).
Group dynamics	Barrier	**External factors and disruptions**: Punctuality issues, absences, holidays, and competing obligations disrupted group rhythm, while new adult figures occasionally disrupted positive group dynamics.	‘(…)If they are on their own with facilitators that they are used to… but if you introduce somebody else who's not known to them, then you find that they hold back’ (Interview—Senior researcher).
		**Interpersonal tensions and conflicts**: Adolescents’ emotional vulnerability and conflicts caused occasional disrespect or disruption during sessions.	‘We had an incident where one child cried during the session, and we had to pause and calm her down’ (Logbook information—facilitator). ‘The snacks that were brought created some unfortunate discussions among some of the young people, where one participant was portrayed as someone who ate a lot of snacks’ (Logbook—facilitator).
		**Uneven participation and engagement**: Varying energy and focus challenged group dynamics; dominant voices have limited others, affecting group atmosphere.	‘One of the groups who had some of the very dominant kids, didn’t engage very well in the assignment …The dominating group challenged some of the others in the group (…), this created a hostile atmosphere that was difficult to moderate’ (Logbook—facilitator).
		**Levels of understanding and facilitation readiness**: Differences in adolescents’ social and emotional backgrounds and experience reduced group cohesion, and less experienced facilitators struggled with engagement and adolescents’ behavior.	‘We noticed some of the teenagers couldn’t express themselves fully in writing. This made it a bit difficult to understand their perspectives and their knowledge regarding some activities’ (Logbook—facilitator).
Partnership	Facilitator	**Pre-existing networks and institutional needs**: Existing collaborations, proximity to influential actors, and alignment with institutional interests increased positive visibility among partners.	‘(…) they were like, ‘oh, that's cool to work together with you, because then you can do that work for us involving inhabitants. And then we don't have to do that anymore’. So for them, it was an advantage to work together with us)’ (Interview—Senior researcher).
		**Trust and credibility**: Positive community perceptions of host organizations and staff appeared to support acceptance of a new project like YoPA.	‘Over the years, the relationship developed and it's quite good now to a point where now the community trusts or believes in whatever [the host organization] is here to deliver’ (Interview—Facilitator).
		**Relationship building**: Establishing positive connections between YoPA representatives (facilitators and researchers) and local implementers (e.g. teachers or youth club staff) turned managing the process easier.	‘The relationship is perfect. Anytime they feel they are having a meeting that they want us to be there, they [YoPA staff] will call me on the phone’ (Interview—External stakeholder). ‘After each YoPA meeting, we sat down and talked about what worked in relation to the young people, and she [facilitator] asked, ‘Is there anything in the way we run the program that you think should be different or would work better?’ (Interview—External stakeholder).
	Barrier	**Institutional dependence**: Although having partner institutions was essential, it also posed challenges, namely when the project’s progress was dependent on these institutions.	‘They just wanted to ask one [institution] at a time. And then that [institution] said no. And then they had to go to another [institution] (…) it went so slow’ (Interview—Senior researcher).
Motivation	Facilitator	**Personal purpose**: Adolescents appeared to join YoPA’s co-creation process with individual goals, such as contributing to community change, gaining experience, or developing leadership and research skills. They seemed to view it as a positive opportunity for personal impact; as well as adult stakeholders.	‘Some [adolescents] wanted to make a contribution to change in their neighbourhood. Others wanted to learn about research schemes or use the certificate for university applications’ (Interview—facilitator & PhD student).
		**Sense of belonging**: Motivation appeared to increase when adolescents felt part of a supportive group that valued their voice, potentially fostering ownership and consistent attendance.	‘(…) they [adolescents] got that sense of ownership and that it was also a recognition of the seriousness they brought into it. That they felt it was important what they brought and therefore also an important project they could come to’ (Interview—external stakeholder).
		**Opportunity seeking**: Youth and especially adult stakeholders showed interest in social and professional opportunities that could arise through YoPA, such as international connections, visibility, or future prospects.	‘We do have networks and connections, (…), we have that proximity to resources and opportunities… being involved (…) puts you in a position that maybe you could be considered for something in the future’ (Interview—senior researcher).
		**Fun**: Enjoyment, novelty, and informal incentives (e.g. games, creative activities, refreshments) seemed to enhance engagement, while the project’s playful and social aspects helped sustain participation.	‘(…) Why do you want to be part of this?’ They [adolescents] told us that they thought it was fun. They thought it was important’ (Interview—PhD student).

#### Experiential

***User-friendly execution***. The identified subthemes mainly captured barriers related to implementing the YoPA co-creation protocol in practice. A key challenge involved translating complex theoretical concepts, such as systems thinking and theories of change, into activities that were accessible and engaging for adolescents. Facilitators frequently needed to simplify or adapt the materials so that adolescents could contribute meaningfully. These experiences highlight the tension between maintaining methodological consistency and adapting methods to diverse contexts. In the logbooks, comments like ‘I believe the complexity of the assignment was too high or difficult’ illustrate this challenge. As a senior researcher observed, ‘these abstract concepts are incredibly difficult to get engagement with, because people are thinking, well, what does that mean? What does that mean to me today?’

Table 2 shows the subthemes and illustrative quotations of the experiential dimension components.

**Table 2:** Detailed subthemes and quotations illustrating components of the dimension experiential.

Experiential			
Component	Category	Identified subthemes	Quotations (e.g.)
User-friendly execution	Barrier	**Translation of theory**: The systems mapping process, grounded on theories of change and systems thinking, proved difficult to translate into accessible, engaging activities for youth. Limited familiarity with these theories further hinder session facilitators and researchers.	‘If we present this back to the youth, they won't engage with it, you know, because it's just insanely complex and all sorts of things going in all sorts of directions (…) So how do we now simplify this so that the youth can actually give their input and contribute (…)’ (Interview—senior researcher).
		**Practical challenges**: The protocol’s structure—restricted participant numbers, time constraints, and the balance between co-development and facilitator-led tasks—aligned with contextual factors reinforced some practical challenges for the sessions’ facilitators.	‘It was still an issue since we had to stick to a particular protocol and there's a time frame and everything. So it actually delayed some activities’ (Interview—facilitator). ‘(…) and maybe the manual for the workshop had to be kind of redesigned for this group [children with specific disabilities] for us to kind of design the assignments to fit them and this group specifically (…)’ (Interview—facilitator).

#### Context

***Contextual influences***. Contextual factors represented one of the most significant sources of barriers across sites. Socioeconomic adversity, including poverty, unsafe environments, and unstable living conditions, often shaped adolescents’ ability to prioritize participation in the sessions. When families were facing urgent daily challenges, involvement in health-related co-creation activities could appear less immediately relevant: ‘the biggest challenge was also working with the vulnerable teenagers because they have so much other stuff going on. And it was so difficult to make YoPA seem important (…)’. Broader structural issues, such as unreliable infrastructure, school schedules, or transport constraints, also affected the organization of sessions. In some settings, however, alignment with political priorities supporting youth participation facilitated collaboration with local authorities.

***Resources and support***. Resources and support were mainly described as facilitators. Access to organizational infrastructure, materials, and stable physical spaces enabled consistent session delivery. In addition, the involvement of local staff such as teachers or youth workers provided continuity and practical assistance during sessions, like seen in a logbook comment: ‘It worked really well that they [adolescents] were given a secretary to assist them—something they themselves highlighted as helpful. Some of them emphasized this, as they face challenges related to dyslexia’. Training and guidance within the consortium also strengthened facilitators’ confidence. Regular meetings and exchanges with experienced researchers supported problem-solving and improved implementation practices. Nevertheless, cross-country collaboration sometimes posed challenges, as geographical distance and differing timelines limited opportunities for shared learning.

***Feasibility***. Feasibility captured barriers related to whether the co-creation process could realistically be implemented within existing constraints. Competing academic schedules, institutional requirements, geographic distance from sessions site and staff workload affected attendance and session planning.

Institutional and bureaucratic processes further influenced feasibility, particularly when integrating the project within school curricula or obtaining necessary approvals. These barriers illustrate how co-creation initiatives depend not only on motivation but also on the practical conditions that enable its process. In one setting, this was a recurring issue: ‘This constant change of students schedules, sometimes even every 2 weeks, makes the planning of the sessions extremely difficult’.

Table 3 shows the subthemes and illustrative quotations of the Context dimension components.

**Table 3:** Detailed subthemes and quotations illustrating components of the dimension context.

Context			
Component	Category	Identified subthemes	Quotations (e.g.)
Contextual influences	Facilitator	**Alignment with political agenda**: Some political/policy partners had youth participation included in their agenda, which made them more interested in YoPA.	‘They have a vision that listening to children is very important. So that's also the way they work (…) in this specific municipality, they really want to listen to the young people and children’ (Interview—Senior researcher).
	Barrier	**Socioeconomic and environmental adversity**: Poverty, violence, unemployment, and unsafe environments appeared to affect young people’s daily lives and their participation in the co-creation process, underscoring the relevance of context-sensitive approaches.	‘People are struggling with unemployment. They are struggling with just having something to eat… So now when you go to them and say, ‘let's work on something that will help you with your physical health or mental health’, it doesn't really come out as important to them (…)’ (Interview—PhD student and facilitator).
		**Systemic fragility and logistical constraints**: Unreliable infrastructure, scarce resources, and disruptions such as power cuts, teacher absences, exams, or transport issues hindered session management.	‘It feels as if they don't have enough infrastructure to support the students. ‘(Interview—PhD student and facilitator). ‘It's a power outage that happens almost every day. So now we need to plan our days based on what time we'll have electricity (…)’ (Interview—PhD student and facilitator).
		**Social disparities and negative personal experiences**: Differences in background, language, and education appeared to limit mutual understanding between adolescents and equitable participation in the co-creation process.	‘How much discipline and violence is in these young people's lives, that their go-to answer if someone needs support to make health changes, (…), is immediately to go to discipline, that often involves forms of violence’ (Interview—Senior researcher). ‘(…) you find that you're dealing with a child who is coming from a poor family, who has to come and interact with other young people who are coming from families that are well off than that particular participant. And then (…) they develop this inferiority complex’ (Interview—PhD student and facilitator).
Resources and support	Facilitator	**Existing organizational structures**: Pre-existing networks and collaborations, along with available physical spaces and materials to organize the sessions turned coordination smoother.	‘The [partner organization] gave us a number of options of [institutions] that when we explained the project to them, they told us some [institutions] that would best fit (…)’ (Interview—senior researcher). ‘We are able to have a space as a permanent office for the YoPA programme. And we have an average security to keep all the materials intact, and even the students’ (Interview—External stakeholder).
		**Relational support**—**allocated staff**: In all sites, staff from local institutions (e.g. teachers, tutors, or youth workers) were assigned to support YoPA. Their involvement was key to ensuring smooth co-creation sessions.	‘We had two staff that were assigned to the projects. They are tutors, like two female tutors that were actually attached to the project (…), they always attend almost every session’ (Interview—facilitator).
		**Training and expertise**: Implementers (e.g. facilitators) benefited from guidance and support from experienced researchers through meetings, tutoring, or group training. Local staff’s prior expertise with youth or the intervention area also most likely supported the process.	‘I also gave them [facilitators] advice on how to present YoPA to teachers, for example, what is important in the presentation.’ (Interview—senior researcher). ‘If the facilitators don't get it, then they can't deliver it. And I think that shift now, that's happened, with the facilitators connecting directly to discuss the implementation on a more pragmatic and practical level on a Monday [in the regular facilitators’ meetings] (…)’ (Interview—senior researcher).
	Barrier	**Cross-learning challenges:** Being a multi-country project made it more difficult to find support across all teams, as processes and timelines were different, plus the geographical distance.	‘(…) in the process, it's not very much aligned (…) it would be nicer if we could have more cross learning during the process’ (Interview—Senior researcher).
Feasibility	Barrier	**Competing priorities and overload**: Youths’ academic demands and busy schedules appeared to limit consistent participation, while local staff overload also constrained co-creation implementation.	‘This constant change of students’ schedules, sometimes even every 2 weeks, makes the planning of the sessions extremely difficult’ (Logbook—facilitator).
		**Geographic and logistical constraints**: Distance between adolescents, facilitators, and intervention sites reduced attendance, ownership, and facilitation capacity.	‘The [institution] is close to your house, it is possible, but in general, they are further away from the centre. So more kids have to bike (…) or use other public transports, for example’ (Interview—Senior researcher).
		**Institutional barriers and curriculum integration**: Bureaucratic delays, limited institutional readiness, and challenges integrating YoPA into school curricula seemed to hinder implementation.	‘Now, I wouldn't say it took so long because (…) bureaucracy, things like that in this part of the world have to go through different people’ (Interview—PhD student and facilitator). ‘We couldn't do it during school hours. So it has to be in a free time. And that is always more difficult (…)’ (Interview—Senior researcher).

#### Impact

***Mechanisms of impact and empowerment***. For the analysis, we merged the components Empowerment and Mechanisms of Impact due to overlapping subthemes. Interviewees described how allowing youth to have their opinions listened to and incorporated into project decisions fostered a sense that ‘their voice matters’, strengthening agency and validation, which appeared to act as mechanisms.

Engagement in collaborative activities also supported the development of self-confidence and leadership skills. Adolescents became more comfortable speaking publicly, expressing their ideas, and supporting peers during discussions. A senior researcher observed that they developed self-trust, ‘self-trust in many things. Self-trust in standing up, hearing your own voice. Self-trust in that you have something to say’. The sessions also created spaces where adolescents could express vulnerability and share personal experiences, fostering trust within the co-creation groups. Peer collaboration further reinforced these outcomes, as they learned from one another and could collectively contribute to initiatives aimed at improving their communities.

Table 4 shows the subthemes and illustrative quotations of the Impact dimension components.

**Table 4:** Detailed subthemes and quotations illustrating components of the dimension impact.

Impact			
Component	Category	Identified subthemes	Quotations (e.g.)
Mechanisms of impact + empowerment	Facilitator	**‘My voice matters’**: Adolescents’ voices and opinions were respected and reflected in project decisions, which fostered validation and agency.	‘I think a space to be heard is a big thing. That sense of what they say [adolescents] is respected and valued. And that what they say can go towards creating something that they will see (…)’ (Interview—Senior researcher).
		**Unleash of inner power**: Youth appeared to recognize their capacity for critical thinking, decision-making, and leadership, awakening a sense of personal potential.	‘It enabled the students to think for themselves and discover some hidden talents in them. Some areas that they even think they are unable or they are not capable. But at the end of the programme, they will see themselves, ‘ah, so I can do this myself’ (Interview—External stakeholder).
		**Building self-confidence**: Engaging in public speaking, facilitation, and leadership roles helped adolescents overcome shyness and strengthen confidence in their ideas and communication.	‘And one major thing that we have pushed for them [adolescents] to do more is speaking out. That means having some level of confidence in themselves and their own ideas to communicate them effectively’ (Interview—Senior researcher).
		**Space for vulnerability**: The co-creation process appeared to foster trust and psychological safety, allowing adolescents to express vulnerability, share insecurities, and navigate cultural or linguistic barriers—becoming a source of connection and growth.	‘(…) There are some young people who may have had a bit of difficulty with it at times in the process. When they see an adult leading it, it has also been more secure’ (Interview—External stakeholder).
		**Peer collaboration**: Within co-creation groups, collaboration potentially promoted collective empowerment, as adolescents learned from and supported each other.	‘They created something for their peers. And (…) I'd say ‘I contributed to the community. I have something that I contributed’, I'd say to my peers’ (Interview—Facilitator).

### Realist-inspired: Context–Mechanism–Outcome configurations

To address the second objective, an exploratory CMO analysis was conducted to examine how contextual conditions and underlying mechanisms interacted to produce the observed outcomes. Only the four overall most frequently reported PROSECO components were analysed as CMO configurations, for conciseness.

#### CMO configurations for the component Group Dynamics and Partnerships

Across settings, the CMOs related to Group Dynamics illustrate how co-creation processes not only depend on structural arrangements like a successful recruitment or external resources, but on subtle interpersonal mechanisms that sparkle in the nuances of the interaction between all actors involved.

These CMOs show that group-based co-creation requires intentionally shaping relational conditions that lower negative experiences and democratize voice. At the same time, facilitators must navigate both universal group processes and context-specific socio-cultural dynamics.

These CMOs highlight that partnership dynamics strongly shape implementation conditions and initial mobilization for co-creation; therefore, partnerships should be highly prioritized when planning the project. From an implementation perspective, it shows that co-creation depends not only on formal partnerships but on the density and quality of relational ties that connect institutions to communities and frontline actors.

Table 5 provides the CMOs related to group dynamics and partnerships.

**Table 5:** CMO configurations related to group dynamics and partnerships.

Category	Context	Mechanism	Outcome
**Group dynamics**			
Facilitator	Where informal relationships were established between the youth and the session facilitators	Feeling of connection and relational bonding; trust	Youth are more open to actively engaging; sessions run smoothly, and the ambiance is more positive.
Facilitator	When session facilitation was structured and based on agreement between adolescents and facilitators	Respect and an increased sense of ownership	Equitable participation, less conflict, more productive sessions
Barrier	When there were either cultural or linguistic gaps in the communication with or between youth	Reduced connection and trust, misinterpretation	Limited participation and lower engagement, weaker sense of ownership, and disinterest might lead to dropout
Barrier	When group cohesion was threatened by power imbalances (e.g. individuals dominated discussions or opinions, differences in age and gender)	Feelings of embarrassment, misalignment, and inferiority; questioning of one´s own legitimacy	Tense or one-sided discussions, shyness, reduced participation, and more interpersonal conflicts
**Partnerships**			
Facilitator	Where there was pre-existing institutional collaboration	Trustworthiness, credibility, and confidence among actors	Faster project initiation, smoother mobilization of local actors, and streamlined decision-making
Facilitator	Where strong partnerships with community-based organizations were in place	Willingness and motivation; sense of shared purpose	High stakeholder engagement, easier recruitment of youth, and stronger legitimacy within the community
Facilitator	Where partners were able to build personal, relationally grounded connections with frontline local actors	Feeling of connection and relational bonding, not just transactional; affective commitment	Goodwill of local actors, smooth and close communication, openness to help and engage
Barrier	Where relying mostly or solely on governmental collaboration happened	Lack of ownership and personal disconnection from frontline key actors	Less engagement, limited commitment from certain actors, or uneven participation

#### CMO configurations for the component contextual influences and resources and support

These CMOs illustrate how contextual influences influence the implementability of co-creation activities and directly shape participation quality and attendance. From an implementation perspective, these insights underline that co-creation cannot be isolated from broader social realities and political readiness. Institutional cooperation, attendance, and engagement reflect not only motivation, but the structural conditions that determine partners’ availability and adolescents’ cognitive and emotional bandwidth.

Across contexts, these CMOs show how implementation quality and consistency in co-creation are deeply shaped by the presence or absence of supportive structures and networks. This shows an important implication for implementation teams: co-creation work relies as much on the relational and infrastructural ecosystem surrounding teams as on the activities themselves, and substantial investment in internal shared learning mechanisms is essential to maintain motivation, cohesion, and innovation across diverse settings.

Table 6 provides the CMOs related to contextual influences and resources and support.

**Table 6:** CMO configurations related to contextual influences and resources and support.

Category	Context	Mechanism	Outcome
**Contextual influences**			
Facilitator	Where the local authority has the participation of youth as part of their political agenda	Sense of alignment and institutional legitimacy; shared ownership of goals	Faster partners buy-in, higher institutional responsiveness, and cooperation
Barrier	Where youth and their families experience structural deprivation	Chronic stress, reduced cognitive bandwidth, and prioritization of immediate needs over participation in co-creation	Lower attention and energy during sessions; inconsistent participation patterns
Barrier	Where home environments were demanding or unsafe (e.g. household labour responsibilities, conflict, or violence)	Heightened emotional strain and protective coping strategies that reduce cognitive and emotional availability	Irregular engagement and reduced readiness to participate, inconsistent participation patterns
**Resources and support**			
Facilitator	In settings with pre-existing collaborative networks, accessible physical spaces, and available materials that supported implementation tasks	Sense of preparedness; reduced stress and coordination load; implementers (e.g. facilitators) feeling supported	Smooth implementation; implementers focus more on session planning; better adherence to timeline; increased confidence
Facilitator	When structured training and support were available within the consortium	Strengthened perceived self-efficacy of facilitators, shared competence, and internal cohesion	Greater internal trust, more consistent implementation quality, and easier access to shared resources and know-how
Barrier	When geographical distance and dispersed implementation sites limit opportunities for cross-learning and collaborative problem-solving	Less exposure to diverse ideas for the project, reduced collective learning, limited common cultural understanding	Different implementation perspectives; missed opportunities for synergy, and joint innovation; fragmented sense of global team identity

## Discussion

This study examined early implementation barriers and facilitators of co-creation within the multi-country YoPA project. We used realist thinking to explore how factors operated across contexts, in order to inform future co-creation initiatives.

The identified barriers and facilitators spanned four PROSECO dimensions but were marked within the Participation dimension. It was clear how Group Dynamics shaped the whole implementation process, which is aligned with literature. In Longworth’s *et al*. (2024b) review, several authors indicated the challenge of ‘effectively engaging with participants deeply entrenched in complex sociocultural contexts. Power shifting, for example, also seems to be an important strategy when working with adolescents, but it can also be hard to handle. Therefore, it is desirable to include stakeholders already used to conducting such processes, especially in Minority countries, where youth can challenge authority figures more often. It is also important to acknowledge that session facilitators’ own identities may influence how dynamics are applied. Involving adult stakeholders in the co-creation process had different challenges in each setting, but availability (either to arrange practical aspects or to actively engage in the process) was a major one. This should be considered when aligning expectations with partners from the beginning, particularly regarding the time and effort required to build the process, as well as arranging logistical aspects.

Regarding Partnership, barriers to their good functioning were related to relationship building and relying too much on the institutional contact persons. Where partnerships were established directly with respected civil organizations, community trust eliminated most of other potential barriers related to this issue. In line with previous co-creation and participatory research studies, our findings demonstrate that relational groundwork is essential in co-creation processes, not merely a ‘nice to have’, given its centrality to key process outcomes. As Smith *et al*. (2023) stated, ‘building trust and sharing power are fluid and fragile processes that are in need of constant negotiation and re-negotiation (p. 169)’. Similarly, Cornish *et al*. (2023), highlighted that this relationship-building must be a constant practice.

This is also consistent with findings from the Kids in Action project (Anselma *et al*. 2020), where the relationship between researchers, children and community partners was an important facilitator. In Jagosh *et al*.'s (2012) realist review, Partnership Synergy was identified as a central mechanism promoting positive outcomes across participatory research studies. In the Danish YoPA team, youth reported group dynamics as something central to their positive experience (Madsen *et al*. 2026).

The component of motivation was identified across all sites and strongly reported by interviewees, although an in-depth analysis of individual motivational drivers was beyond the scope of this study. From the perspective of session facilitators and adult stakeholders, youth motivation appeared to stem from multiple sources, including opportunities for skill development (e.g. CV building among older adolescents), tangible incentives such as refreshments, engaging and enjoyable activities, and the creation of a space where young people felt heard. Adult stakeholders’ motivation was primarily linked to purpose-driven engagement, opportunity seeking, and the development of partnerships for future initiatives. These findings align with Self-Determination Theory (Deci and Ryan 2000), suggesting that motivation was fostered through the satisfaction of autonomy (having a voice and influencing decisions), competence (developing skills and gaining recognition), and relatedness (feeling part of a valued group). Overall, sustained participation was more likely when both youth and adults perceived personal relevance and a sense of belonging, reinforcing the importance of designing co-creation processes that support basic psychological needs.

The recruitment component of the PROSECO framework, although not notable in the analysis and therefore not included in the main Results, warrants attention given its central role in setting the base for the co-creation process. Recruitment approaches varied across countries, yet success consistently depended on strong collaboration with local partners, closely linking this component to Partnership. In school contexts, recruitment was most effective when led by teachers or other trusted adults, whereas in community settings it worked best through established institutional networks such as youth clubs or NGOs. Barriers commonly reported included limited interest in the topic, competing adolescent priorities, challenges in securing parental consent across contexts, and communication gaps. Most teams characterized recruitment as resource-intensive, requiring sustained time and effort.

Notably, recruitment appeared more challenging and slower in Minority country settings, where YoPA competed with a range of existing youth programmes and activities and could have been initially perceived as ‘one more programme.’ In contrast, in Majority countries settings, YoPA was more easily positioned as distinct and valuable, particularly as an internationally funded initiative linked to recognized academic institutions, which facilitated engagement.

Woelk's (1992) lens on patient and community-centred work in health can support a critical analysis on this topic. He observed that engagement was heavily conditioned by structural context, such as broader economic conditions, institutional arrangements, and power relations rather than by programme design alone. This suggested that participation is filtered through culturally embedded perceptions of programmes and is more readily mobilized when initiatives are perceived as gateways to scarce resources and external recognition, which may explain why YoPA could have been seen by youth in high-income contexts as ‘another programme’ rather than a distinctive opportunity. As Woelk cautions, apparent ease of participation in lower-resource contexts does not necessarily equate to empowerment, as engagement may also reflect structural scarcity and unequal power relations rather than sustained community ownership.

The fact that the component of contextual influences represented relevant barriers to the co-creation process aligns with the realist approach, that highlights the role of the context when analysing how the mechanisms operate.

Shaw *et al*. (2018) discuss the complexity of the context concept within realist-informed approaches and argue that grouping contextual influences into a few categories and linking them directly to mechanisms risks oversimplification. Instead, they propose examining networks of multiple interacting influences and considering how some contextual factors may also operate as mechanisms. In this sense, the PROSECO framework’s context dimension can support a deeper understanding of co-creation settings, but its components should be used as guiding constructs rather than fixed categories.

Another barrier mentioned across countries concerned the application of the systems approach of the co-creation protocol. Facilitators and senior researchers consistently reported that implementing systems-thinking tasks, such as developing systems maps and engaging with abstract theoretical concepts, was challenging with both adolescent and adult stakeholders, though the difficulty was more frequently reported by researchers in some sites than others. This challenge of complexity was also observed in Heinze *et al*. (2025), who conducted a similar process with both young students and adults from vocational schools. This highlights a broader gap: clearer guidance is needed on how to translate systems theory into feasible, youth-friendly practice within co-creation processes. Despite the participatory orientation of the project, researchers often had to refine and reinterpret the systems maps using their own expertise and resources. Mechanisms involving the execution of this component must be further analysed, as well as the specific outcomes that applying such concepts may have for the experience of young people in the process.

Co-creation must be guided by working principles, like the ones described in Smith *et al*. (2023) (e.g. reciprocity, diversity, equitable partnerships) as well as in Chastin *et al*. (2025). (e.g. inclusive, flexible, actionable, and collectively owned). The potential mechanisms leading to positive outcomes as seen in this analysis, reinforce that adequate resources, power sharing, pluralism, relational bonding, are all crucial for an ethical and effective co-creation process. Expanding the understanding about how these mechanisms operate in different contexts can support the implementation of co-creation processes.

### Strengths and limitations

The integration of a framework-driven thematic analysis with a realist-informed approach is a key strength of this study. The use of multiple data sources and the involvement of researchers from both African and European countries further enhance the credibility and contextual relevance of the findings.

Applying the PROSECO Framework presented both opportunities and challenges. Although designed for evaluating co-creation processes, no prior empirical applications were identified at the time of analysis, positioning this work as an early contribution to its methodological development.

The framework offered a comprehensive and adaptable structure for examining co-creation. While its many components may pose analytical challenges, this study addressed overlaps by identifying subthemes within the existing framework, improving coherence and analytical depth. This approach enhances transparency and supports replication in future studies. The integration of Context–Mechanism–Outcome configurations further strengthened the analysis by revealing how contextual factors influence mechanisms shaping implementation outcomes.

Adolescents were not interviewed for this study. Including direct youth perspectives, which is planned in future YoPA studies, will provide valuable complementary insights into the barriers and facilitators of the YoPA co-creation process. As interviews were conducted post-implementation, some recall bias may have occurred, although triangulating data sources likely mitigated this. The logbook data may also be subject to bias, as the prompts primarily encourage reflection on the dynamics of the sessions. Although they allow some discussion of contextual factors, they do not explore these in depth.

Further development of detailed CMO configurations and exploration of alternative analytical strategies could enhance theory testing and guide the use of the PROSECO Framework across diverse contexts.

## Conclusion

This study shows that the first co-creation phase in the multi-country YoPA project was shaped by group dynamics, partnerships, motivation, contextual influences, access to resources and the challenging use of systems thinking. The findings highlight the importance of shared power, cultural adaptation, and strong local collaboration for ethical and effective co-creation across diverse contexts. They also suggest that future co-creation initiatives tackling health-related issues should include clear guidance, adequate resources, and youth-friendly methods to support meaningful participation and implementation.

## Supplementary Material

daag134_Supplementary_Data

## Data Availability

The data underlying this article cannot be shared publicly due to participant privacy concerns and the impossibility of complete anonymization. The data may be shared on reasonable request to the corresponding author.

## References

[daag134-B1] Agnello DM, Anand-Kumar V, An Q et al Co-creation methods for public health research—characteristics, benefits, and challenges: a Health CASCADE scoping review. BMC Med Res Methodol 2025;25:60. 10.1186/s12874-025-02514-4 40050729 PMC11884017

[daag134-B2] Anieto EM, Dall PM, Abaraogu U et al The effectiveness of co-created lifestyle interventions in improving health behaviour, physical and mental health in adults with non-communicable diseases: a systematic review with meta-analysis. Public Health 2025;248:105929. 10.1016/j.puhe.2025.105929 40845709

[daag134-B3] Anselma M, Chinapaw M, Altenburg T. Not only adults can make good decisions, we as children can do that as well: evaluating the process of the youth-led participatory action research ‘Kids in Action’. Int J Environ Res Public Health 2020;17:625. 10.3390/ijerph17020625 31963706 PMC7014142

[daag134-B4] Anyon Y, Bender K, Kennedy H et al A systematic review of youth participatory action research (YPAR) in the United States: methodologies, youth outcomes, and future directions. Health Educ Behav 2018;45:865–78. 10.1177/1090198118769357 29749267

[daag134-B5] Baum F, MacDougall C, Smith D. Participatory action research. J Epidemiol Community Health 2006;60:854–7. 10.1136/jech.2004.028662 16973531 PMC2566051

[daag134-B6] Biddle SJH, Ciaccioni S, Thomas G et al Physical activity and mental health in children and adolescents: an updated review of reviews and an analysis of causality. Psychol Sport Exerc 2019;42:146–55. 10.1016/j.psychsport.2018.08.011

[daag134-B7] Bonell C, Prost A, Melendez-Torres GJ et al Will it work here? A realist approach to local decisions about implementing interventions evaluated as effective elsewhere. J Epidemiol Community Health 2020;75:46–50. 10.1136/jech-2020-214287 32907917

[daag134-B8] Brand SL, Quinn C, Pearson M et al Building programme theory to develop more adaptable and scalable complex interventions: realist formative process evaluation prior to full trial. Evaluation 2019;25:149–70. 10.1177/1356389018802134

[daag134-B9] Braun V, Clarke V. Using thematic analysis in psychology. Qual Res Psychol 2006;3:77–101. 10.1191/1478088706qp063oa

[daag134-B10] Chastin SFM, Smith N, Agnello DM et al Principles and attributes of evidence-based co-creation: from naïve praxis toward a trustworthy methodology—a health CASCADE study. Public Health 2025;248:105922. 10.1016/j.puhe.2025.105922 40992098

[daag134-B11] Chinapaw MJM, Klaufus LH, Oyeyemi AL et al Youth-centred participatory action approach towards co-created implementation of socially and physically activating environmental interventions in Africa and Europe: the YoPA project study protocol. BMJ Open 2024;14:e084657. 10.1136/bmjopen-2024-084657 PMC10882351 38387985

[daag134-B12] Cornish F, Breton N, Moreno-Tabarez U et al Participatory action research. Nat Rev Methods Primers 2023;3:34. 10.1038/s43586-023-00214-1

[daag134-B13] Dalkin SM, Greenhalgh J, Jones D et al What’s in a mechanism? Development of a key concept in realist evaluation. Implement Sci 2015;10:49. 10.1186/s13012-015-0237-x 25885787 PMC4408605

[daag134-B14] Dalkin SM, Hardwick RJL, Haighton CA et al Combining realist approaches and normalization process theory to understand implementation: a systematic review. Implement Sci Commun 2021;2:68. 10.1186/s43058-021-00172-3 34174966 PMC8234627

[daag134-B15] Deci EL, Ryan RM. The “what” and “why” of goal pursuits: human needs and the self-determination of behavior. Psychol Inq 2000;11:227–68. 10.1207/S15327965PLI1104_01

[daag134-B16] Frank KI . The potential of youth participation in planning. J Plan Lit 2006;20:351–71. 10.1177/0885412205286016

[daag134-B17] Gibbs A, Campbell C, Maimane S et al Mismatches between youth aspirations and participatory HIV/AIDS programmes in South Africa. Afr J AIDS Res 2010;9:153–63. 10.2989/16085906.2010.517482 25860524

[daag134-B18] Greenhalgh T, Jackson C, Shaw S et al Achieving research impact through co-creation in community-based health services: literature review and case study. Milbank Q 2016;94:392–429. 10.1111/1468-0009.12197 27265562 PMC4911728

[daag134-B19] Hale GE, Colquhoun L, Lancastle D et al Review: Physical activity interventions for the mental health and well-being of adolescents—a systematic review. Child Adolesc Ment Health 2021;26:357–68. 10.1111/camh.12485 34105239

[daag134-B20] Halvorsrud K, Kucharska J, Adlington K et al Identifying evidence of effectiveness in the co-creation of research: a systematic review and meta-analysis of the international healthcare literature. J Public Health 2021;43:197–208. 10.1093/pubmed/fdz126 PMC8042368 31608396

[daag134-B21] Heinze C, Klinker CD, Sidenius A et al Process evaluation of a participatory systems approach to promote health and well-being among students at vocational schools. Health Educ Res 2025;40:cyaf022. 10.1093/her/cyaf022 40448680 PMC12125708

[daag134-B22] Howlett N, Fakoya O, Bontoft C et al A realist evaluation of community champion and participatory action approaches during the COVID-19 pandemic. Front Public Health 2024;12:1355944. 10.3389/fpubh.2024.1355944 38939557 PMC11208485

[daag134-B23] Jackson SF, Kolla G. A new realistic evaluation analysis method: linked coding of context, mechanism, and outcome relationships. Am J Eval 2012;33:339–49. 10.1177/1098214012440030

[daag134-B24] Jagosh J . Realist synthesis for public health: building an ontologically deep understanding of how programs work, for whom, and in which contexts. Annu Rev Public Health 2019;40:361–72. 10.1146/annurev-publhealth-031816-044451 30633712

[daag134-B25] Jagosh J, Macaulay AC, Pluye P et al Uncovering the benefits of participatory research: implications of a realist review for health research and practice. Milbank Q 2012;90:311–46. 10.1111/j.1468-0009.2012.00665.x 22709390 PMC3460206

[daag134-B26] Lemire S, Kwako A, Nielsen SB et al What is this thing called a mechanism? Findings from a review of realist evaluations. New Dir Eval 2020;2020:73–86. 10.1002/ev.20428

[daag134-B27] Longworth GR, Agnello DM, Chastin S et al Evaluating the co-creation process in public health interventions: the PROSECO framework. Public Health 2025;245:105783. 10.1016/j.puhe.2025.105783 40449476

[daag134-B28] Longworth GR, Erikowa-Orighoye O, Anieto EM et al Conducting co-creation for public and middle-income countries: a systematic review and key informant perspectives on implementation barriers and facilitators. Global Health 2024b;20:9. 10.1186/s12992-024-01014-2 38233942 PMC10795424

[daag134-B29] Longworth GR, Goh K, Agnello DM et al A review of implementation and evaluation frameworks for public health interventions to inform co-creation: a Health CASCADE study. Health Res Policy Syst 2024a;22:39. 10.1186/s12961-024-01126-6 38549162 PMC10976753

[daag134-B30] Madsen L, Westerskov Dalgas B, Pawlowski CS. Understanding co-creation processes with adolescents in YoPA Denmark. Int J Adolesc Youth 2026;31:2608769. 10.1080/02673843.2025.2608769

[daag134-B31] Marchal B, Van Belle S, Van Olmen J et al Is realist evaluation keeping its promise? A review of published empirical studies in the field of health systems research. Evaluation 2012;18:192–212. 10.1177/1356389012442444

[daag134-B32] Messiha K, Altenburg TM, Schreier M et al Enriching the evidence base of co-creation research in public health with methodological principles of critical realism. Crit Public Health 2024;34:1–19. 10.1080/09581596.2024.2371323

[daag134-B33] Pawson R, Tilley N. Realistic Evaluation. London: Sage Publications Ltd., 1997.

[daag134-B34] Renmans D, Sarkar N, Van Belle S et al Realist evaluation in times of decolonising global health. Int J Health Plan Manag 2022;37:37–44. 10.1002/hpm.3530 35647898

[daag134-B35] Rodriguez-Ayllon M, Cadenas-Sánchez C, Estévez-López F et al Role of physical activity and sedentary behavior in the mental health of preschoolers, children and adolescents: a systematic review and meta-analysis. Sports Med 2019;49:1383–410. 10.1007/s40279-019-01099-5 30993594

[daag134-B36] Rycroft-Malone J, Seers K, Eldh AC et al A realist process evaluation within the Facilitating Implementation of Research Evidence (FIRE) cluster randomised controlled international trial: an exemplar. Implement Sci 2018;13:138. 10.1186/s13012-018-0811-0 30442165 PMC6238283

[daag134-B37] Sawyer SM, Afifi RA, Bearinger LH et al Adolescence: a foundation for future health. The Lancet 2012;379:1630–40. 10.1016/S0140-6736(12)60072-5 22538178

[daag134-B38] Shamrova DP, Cummings CE. Participatory action research (PAR) with children and youth: an integrative review of methodology and PAR outcomes for participants, organizations, and communities. Child Youth Serv Rev 2017;81:400–12. 10.1016/j.childyouth.2017.08.022

[daag134-B39] Shaw J, Gray CS, Baker GR et al Mechanisms, contexts and points of contention: operationalizing realist-informed research for complex health interventions. BMC Med Res Methodol 2018;18:178. 10.1186/s12874-018-0641-4 30587138 PMC6307288

[daag134-B40] Smith B, Williams O, Bone L et al Co-production: a resource to guide co-producing research in the sport, exercise, and health sciences. Qual Res Sport Exerc Health 2023;15:159–87. 10.1080/2159676X.2022.2052946

[daag134-B41] United Nations Children’s Fund. *Investing in a safe, healthy and productive transition from childhood to adulthood is critical*. 2019. https://data.unicef.org/topic/adolescents/overview/

[daag134-B42] Vargas C, Whelan J, Brimblecombe J et al Co-creation, co-design, co-production for public health—a perspective on definition and distinctions. Public Health Res Pract 2022;32:3222211. 10.17061/phrp3222211 35702744

[daag134-B43] Verloigne M, Altenburg T, Cardon G et al Making co-creation a trustworthy methodology for closing the implementation gap between knowledge and action in health promotion: the Health CASCADE project. Perspect Public Health 2023;143:196–8. 10.1177/17579139221136718 37589328

[daag134-B44] Verloigne M, Chastin S, An Q et al The pitfalls of co-creation: reflections from Health CASCADE facilitators on critical events, consequences and preventive and mitigating strategies. Public Health 2025;248:105956. 10.1016/j.puhe.2025.105956 40961857

[daag134-B45] Willis CE, Reid S, Elliott C et al A realist evaluation of a physical activity participation intervention for children and youth with disabilities: what works, for whom, in what circumstances, and how? BMC Pediatr 2018;18:113. 10.1186/s12887-018-1089-8 29544462 PMC5856004

[daag134-B46] Woelk GB . Cultural and structural influences in the creation of and participation in community health programmes. Soc Sci Med 1992;35:419–24. 10.1016/0277-9536(92)90334-M 1519094

[daag134-B47] World Health Organization . Mental health of adolescents. 2025, September 1. https://www.who.int/news-room/fact-sheets/detail/adolescent-mental-health

[daag134-B48] World Health Organization . *Global status report on physical activity 2022*. 2022. https://www.who.int/publications/i/item/9789240059153.

[daag134-B49] World Health Organization Regional Office for Europe. *WHO European regional obesity report 2022*. 2022. https://www.who.int/europe/publications/i/item/9789289057738

