# Supplementary material for: A realist inspired barriers and facilitators evaluation of the co-creation process in the Youth-centred Participatory Action Project
Source: Health Promot Int. 2026 Sep 10;41(5):daag134. doi: 10.1093/heapro/daag134 (PMC13560076; doi:10.1093/heapro/daag134)
Supplement: Supplementary file 1 [file daag134_supplementary_data.zip › Sup._Material_5._Audit_Trail_of_the_Data_Analysis.docx]

### **Supplementary material 5 - Audit Trail of the Data Analysis Process (2025)**

The analytical PROSECO framework was published on May 30 and first accessed by the first author on June 3, initiating familiarization with its components.

On June 25, the framework was shared with three potential coders. An initial meeting on July 4 established that each coder would independently code one interview, followed by a discussion of framework application. During a subsequent meeting on July 15, differences in interpretation emerged, highlighting the need for further clarification of several codes.

In response, an extended version of the codebook (version 2.0) was developed on July 23. This included additional guidance on code application and practical examples, aiming to support shared coding heuristics. Two additional interviews were then coded using pre-defined text segments to facilitate a more objective assessment of inter-coder agreement while minimizing unitization challenges.

A review meeting on July 30 identified continued ambiguities in specific codes (e.g., *Reach*, *Feasibility*, and *User-friendly execution*), leading to further refinements of the codebook. Due to limited availability of one coder, it was decided on August 4 to proceed with two coders only (MFS and CSS).

A second round of coding was conducted on August 6, prioritizing the assignment of a single primary code per excerpt. Initial inter-coder agreement, calculated on August 7 using MAXQDA, was 54.3%, increasing to 60% after correction of coding errors.

Following a consensus meeting on August 12, where disagreements were discussed and shared interpretations refined, agreement increased to 66%. A final meeting on September 8 led to additional consensus decisions, further clarification of selected codes, and correction of double-coding instances. The final inter-coder agreement reached 71.2%.

### ***Additional Notes***

The first author also engaged in four meetings with a junior researcher using the same framework (June–August 2025), which contributed to ongoing clarification of coding practices and analytical strategies.
