# Supplementary material for: A realist inspired barriers and facilitators evaluation of the co-creation process in the Youth-centred Participatory Action Project
Source: Health Promot Int. 2026 Sep 10;41(5):daag134. doi: 10.1093/heapro/daag134 (PMC13560076; doi:10.1093/heapro/daag134)

Supplementary material 1 - Figures with detailed frequency of identified themes for PROSECO components across sites

Fig. 1. Frequency of coded segments by component, per PROSECO dimension.

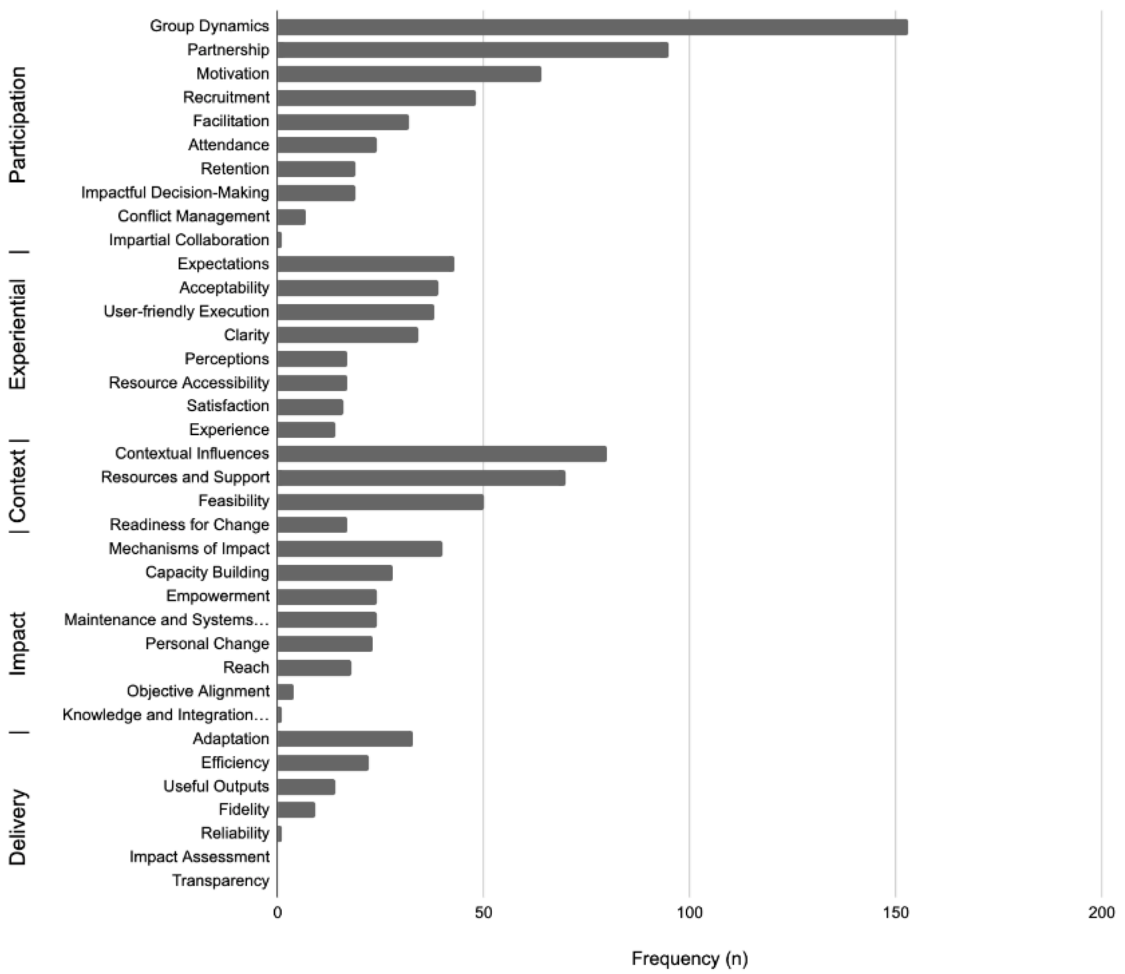

Fig 2. Frequency distribution of the most frequently observed components identified as facilitators.

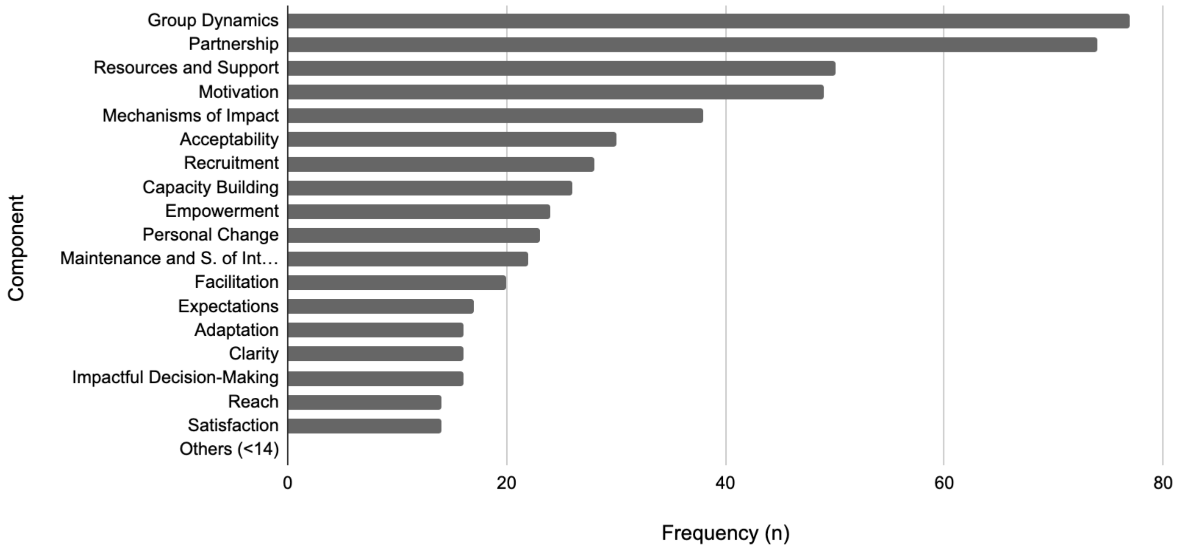

Fig. 3. Frequency distribution of the most frequently observed components identified as barriers.

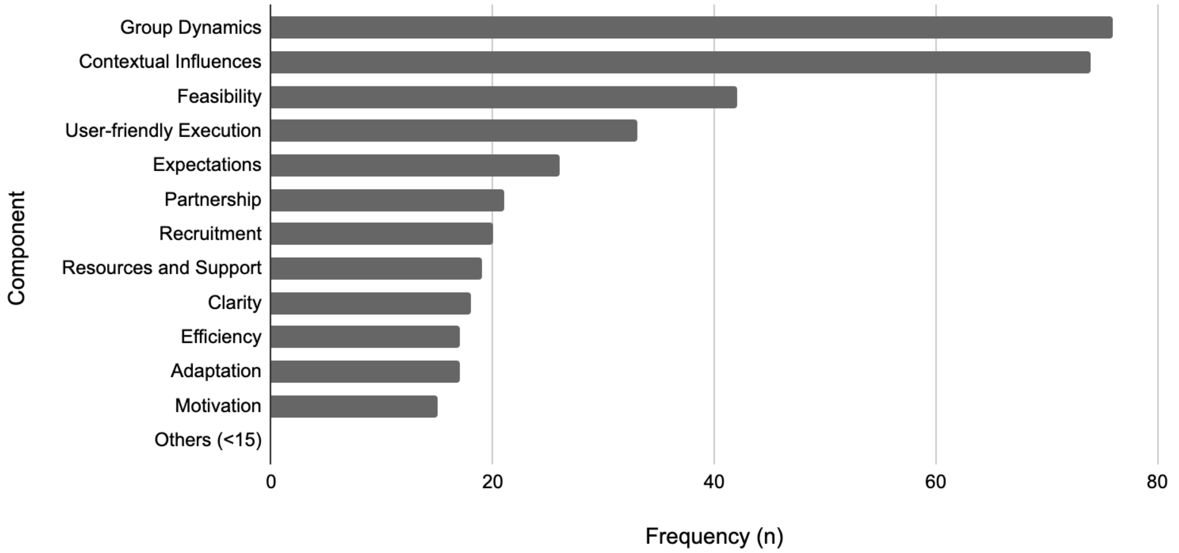

Supplement: Supplementary file 1 [file daag134_supplementary_data.zip › Sup_Material_1_Figures_.pdf]
