# Supplementary material for: A realist inspired barriers and facilitators evaluation of the co-creation process in the Youth-centred Participatory Action Project
Source: Health Promot Int. 2026 Sep 10;41(5):daag134. doi: 10.1093/heapro/daag134 (PMC13560076; doi:10.1093/heapro/daag134)
Supplement: Supplementary file 1 [file daag134_supplementary_data.zip › Sup_Material_2_Semi_structured_interview_guide_.docx]

**Supplementary material 2 - Semi-structured interview guide**

*(Not all topics were addressed for all interviewees. Questions were addressed depending on each interviewee role - “All”, “PhD students”, “Senior researchers”, “Facilitators” or “External stakeholders”)*

**General**

1. Profile and role in the project
2. How were the partnerships made / prioritised and why?

**Intervention**

1. What was different as planned in setting up the project and Phase 1 sessions?
2. Did the first steps of the implementation (recruitment, partnership, logistics) occur as planned? Can you please elaborate?
3. What do you think were the main factors that influenced the setting up and the Phase 1 sessions?
4. What went well / highlights from implementation and first co-creation sessions?
5. How did you experience the process?

**Context**

1. Briefly describe the context in which YoPA was implemented in your setting
2. What do you think is unique about this context?
3. How do you think the context played a role in the implementation of YoPA? To which extent…
4. What were, if any, characteristics of the context that you did not anticipate / presented a surprise element to the process?

**Actors**

1. Briefly describe the target group in your setting
2. How was recruitment made?
3. How did the youth benefit (or not) from the co-creation process?
4. How did the stakeholders benefit (or not) from the co-creation process?
5. How did other community members (who) benefit (or not) from the co-creation process?
6. What were the main factors that helped youth/stakeholders/community members to be involved?
7. What was the role of the partners in your context? Was it accomplished and why?
8. What were the barriers for their involvement and participation?
9. How was your relationship with the YoPA facilitator/staff..

**Mechanisms**

1. How do you think co-creation triggers individual and collective agency? Can you exemplify based on what you observed from the first sessions?
2. What can prevent this from being triggered? What did prevent it from being triggered considering the Phase 1 sessions?

**Outcomes**

1. What are the main outcomes from the first co-creation sessions with youth?
2. How do you think they will affect the next phase of the project?
3. What are barriers and challenges to reaching that / those outcome(s)?
4. Were there any other specific outcomes / unintended from the sessions that you did not expect?

**Other questions**

1. Is there anything you would like to add about the first co-creation sessions of the YoPA project?
