# Supplementary material for: A realist inspired barriers and facilitators evaluation of the co-creation process in the Youth-centred Participatory Action Project
Source: Health Promot Int. 2026 Sep 10;41(5):daag134. doi: 10.1093/heapro/daag134 (PMC13560076; doi:10.1093/heapro/daag134)
Supplement: Supplementary file 1 [file daag134_supplementary_data.zip › Sup_Material_3_Logbook_template_.docx]

**Supplementary material 3 - logbook template**

**General session information**

**Phase: One/Two (mark the option in yellow)**

**Action Team ID/number:**

**Session Number:**

**Session topic:**

**Date:**

**Facilitator(s) name(s):**

**Other adults participating in the session: Nº (	)	Names and Roles:**

**Start time:**

**End time:**

**Where was this session conducted?** (Specific Location - e.g. outdoor/indoor/auditorium/football field, etc.)

**Number of participants expected Nº (	) and who attended the session Nº (	)**

**Reason for non-attendance or any dropout (if known):**

**Participants attending for the first time: Nº (	)**

**Detailed session plan & adaptations**

Provide the detailed plan you prepared for the session and the specific activities (including energizers, capacity building, etc.). Indicate the name of the activity/ies in which the adaptations were made, **which** specific adaptations were made and **why** adaptations were needed.

**Session objective (s):**

**Information of Activities**

*Instructions: Highlight the (Y)* ***or*** *(N) in yellow*

| **Activity 1 name:**  **Did it happen? - (Y) (N)** | **Was any adaptation made? ( Y ) ( N )**  **Specify:**  **Reason for the adaptation:** |
| --- | --- |
| **Activity 2 name:**  **Did it happen? - (Y) (N)** | **Was any adaptation made? ( Y ) ( N )**  **Specify:**  **Reason for the adaptation:** |
| **Activity 3 name:**  **Did it happen? - (Y) (N)** | **Was any adaptation made? ( Y ) ( N )**  **Specify:**  **Reason for the adaptation:** |
| *Add more accordingly* |  |

**Summary of the results of the session**

*Instructions: Highlight the (Y)* ***or*** *(N) in yellow*

**Were the session objectives achieved? ( Y )	( N )	(Partially)**

Why? Please give more details:

**Did everyone participate? ( Y )	( N )	( Partially )**

Please give more details, if applicable:

**Summary of contextual information (other field notes)**

Provide a summary of any contextual information that might influence the process of participatory design. E.g., emotions, conflicts, events, etc.

Potential topics: Challenges and success factors in running the session, both from the group as well as external; specific comments about the participation of youth and stakeholders; potential reasons for most and less engaging participants in the session; distractions and interruptions within and outside the group; specific positive or negative participants behaviour; how was the group interaction and relationship with facilitators; etc.

**Were there any specific needs, unexpected insights or findings from this session?**

If so, please give more details.

Additionally, you can add any specific reflection notes about things you learned as a facilitator in this session, and what you think you could do differently for the next ones, if anything.

**Provide some of the feedback given by the participants to this session**

(highlights of what they liked the most and improvement suggestions)

**Highlights:**

**1.**

**2.**

**3.**

**Improvements:**

**1.**

**2.**
