# Supplementary material for: A realist inspired barriers and facilitators evaluation of the co-creation process in the Youth-centred Participatory Action Project
Source: Health Promot Int. 2026 Sep 10;41(5):daag134. doi: 10.1093/heapro/daag134 (PMC13560076; doi:10.1093/heapro/daag134)
Supplement: Supplementary file 1 [file daag134_supplementary_data.zip › Sup_Material_4_Coding_book_PROSECO_.docx]

**Supplementary material 4 - Coding book for thematic analysis with the PROSECO elements**

**How to read and use this document:**

1. When reading the official definition of the component, pay a closer attention to the highlighted parts, as they reveal what is most important about this component.
2. Read all the **comments** in the third column - they were added only in a few components for extra clarity, or when there is an overlap.
3. Use the examples as a guide, but not literally, as the nuances of the interview transcripts may not be completely reflected in them. Try to find commonalities in the essence of the examples, in the quotes you are coding.

| **COMPONENT** | **OFFICIAL DEFINITION** (Longworth et al., 2025) | **COMMENTS AND EXAMPLES**  **(for coding)** |
| --- | --- | --- |
| **PARTICIPATION** | | |
| 1. Partnership | This component evaluates the collaborations between co-creators in achieving common goals. It also assesses the social bonds and networks of interpersonal and interinstitutional links developed through the co-creation process or implementation of the co-created solution. It may reflect perceptions of trust and mutual understanding between co-creators or stakeholders, including the sharing and agreeing of common goals. | **Use this code whenever the text mentions the partnership directly, as the main reason for success/failure.**  E.g.:  “The trust between our team and the local council grew over time, which helped us agree on the project goals.”  “I think that the staff was very important because we needed them to have the trust of the youth, if that makes sense. So, we really needed their skills and their knowledge of the teenagers. And I think it has been a very good relationship”. |
| 2. Motivation | This component evaluates the underlying reasons for why co-creators engage in co-creation; uncovering what stimulates and energizes them to act. For instance, the reasons they are engaging, decision to join the process, or reasons for staying engaged throughout the process. | **This code should be used when the text is directly related to the engagement of the people involved/mentioned.**  E.g.:  “I joined this project because I wanted to see real change in my community.”  “What kept me going was knowing my input could actually make a difference.” |
| 3. Retention | This component evaluates the degree to which, and ability to, maintain adequate levels of participation throughout the co-creation process. | E.g.:  “I think the fact that the young people are still coming, that we've got that retention and that buy-in, I think that's a major win.”  “Some people dropped out, but the majority of participants played an active role until the end.” |
| 4. Attendance | This component evaluates the presence of co-creators throughout the co-creation process. | E.g.:  “I made sure to be at every workshop so I wouldn’t miss any discussions.”  “Sometimes they couldn’t attend all the meetings for personal reasons, like family or school commitments.” |
| 5. Recruitment | This component evaluates the strategies employed to attract and enlist co-creators in the process. It evaluates the extent to which recruitment has achieved its intended goals and predetermined objectives. If this is among recruitment's objectives, it may involve assessing the extent to which representativeness and diversity among co-creators was attained. | **Use this code whenever the text is talking specifically about recruitment strategies and activities - from any of the involved parts.**  E.g:  “We reached out to them through local community groups, which made it easy to get people involved.”  “I think they did well bringing in people from different backgrounds to the group.” |
| 6. Facilitation | This component evaluates the supporting, and enabling the smooth execution and interaction within the process. It focuses specifically on the facilitator's ability to engage participants, foster inclusive dialogue and ensure equitable contribution among all stakeholders. This might include evaluating how well the facilitator navigates power dynamics, provides useful resources, stimulates creative thinking and manages conflict, contributing to a productive and collaborative co-creation environment. | E.g.:  “The facilitator always made sure everyone had a chance to speak.”  “They helped guide the conversation so it didn’t get off track. And I think that's because our facilitators are so well connected within that group and trusted within them. ” |
| 7. Conflict Management | This component evaluates the ability to address, manage, and resolve conflicts among competing interests within the group. It assesses how well the method or process not only handles conflicts but also leverages them to stimulate constructive debate and foster further development. The emphasis is on promoting resolution in a manner that cultivates a harmonious environment, sustains group cohesion, and utilizes conflicts as opportunities for growth. It aims to ensure that conflicts are handled constructively, and decisions are reached through consensus, thus promoting a cooperative and positive development process. | E.g.:  “When there was a discussion, we would sit together and talk through it with them, reminding them about the project values.”  “There was some tension about funding, but they found a way for everyone to feel heard.” |
| 8. Group Dynamics | This component evaluates the ability to foster active engagement, high involvement, and the strategic use of personalized and interactive aspects to create a harmonious and productive co-creation environment. The focus is on how the method or process manages the dynamics of the group (e.g. empathy,  discussions, trust building) and encourages respectful interactions and cooperation  among co-creators. | **This code should be used when the text refers to dynamics of any “groups”, either at institutional level (e.g. the organization staff and the facilitators) or interpersonal level (e.g. the youth and the facilitators)**  E.g.:  “The team felt comfortable sharing ideas without judgment because there was a safe space.”  “We learned how to listen and build on each other’s thoughts.”  “You know, if they didn't feel a part of the project, they didn't want to accept it.” |
| 9. Impactful decision-making | This component evaluates how well the co-creation method or process facilitates a comprehensive and productive decision-making process. It evaluates whether a decision-making process is structured enough to reach significant,  influential or effective results, that is based on agreement among the co-creators. | E.g.:  “We realized that strategy was not working anymore, and, after discussing it with the teachers, we decided to change how things were done.”  “Participants were listened to several times before the decision was made, so everyone’s voice mattered when we agreed on the final intervention.” |
| 10. Impartial collaboration | This component places a strong emphasis on conducting the co-creation process independent of outside interference. It evaluates the commitment to maintaining a fair and unbiased environment where collaboration occurs without undue influence or bias from internal or external pressures. It aims to ensure that the co-creation process remains focused on the co-creators and their perspectives rather than being swayed by other interests or pressures. | E.g.:  “Even though the municipality had an important role in the project, they gave us complete autonomy to intervene with the community.”  “It didn’t feel like any outside group was influencing what we were doing.” |
| **EXPERIENTIAL** | | |
| 11. Acceptability | This component evaluates the extent to which the co-creation process and method is perceived as suitable and agreeable by the co-creators and the rest of stakeholders involved. It measures how well the preferences, needs, expectations and standards set by co-creators and stakeholders are met, making it suitable for adoption or implementation. | E.g.:  “This method worked well for us — it suited our community’s way of working.”  “I think it was a problem from the beginning because one of the directors did not see value in the activities and we could not convince him to accept the program in that way.” |
| 12. Resource accessibility | This component evaluates the ease with which co-creators can obtain essential resources, including materials, information, digital resources, time, and access to experts. The primary focus is on guaranteeing that all co-creators have impartial and balanced access to vital resources, promoting overall inclusivity and effectiveness. The goal is to achieve  enabling co-creators and facilitators to readily obtain and utilize required resources. | **Use this code whenever the text refers to specific resources made available by the intervention process, as highlighted in the definition.**  E.g.:  “We had access to all the materials and info we needed from day one.”  “In those monthly meetings, they would receive either the support or training they needed to be able to overcome the challenges on the ground and that worked perfectly.” |
| 13. Clarity | This component evaluates the effectiveness in articulating and ensuring a clear understanding of all the collaboration process among the co-creators, including the nature and scope of the task. | **Use this code whenever the text refers specifically to the comprehension (or the lack of) about the process or anything related to the project, from any of the involved parts.**  E.g.:  “They explained what we needed to do really clearly. I always knew what was expected of me at each stage.”  “I think communicating or trying to communicate to them what they're getting out of it is really critical. Sometimes it is hard for stakeholders to get what they are really getting out of this.” |
| 14. Expectations | This component evaluates the extent to which the co-creators' anticipated outcomes, preferences, needs, or expectations were met. | E.g.:  “It turned out the fact she was very far away was a bigger problem than we first thought it would be, because the distance made it more difficult to collaborate.”  “At first, I expected more support, but overall, it met my expectations.” |
| 15. Perceptions | This component evaluates the subjective viewpoints, interpretations, and insights of the co-creators regarding various aspects of the collaborative process and its outcomes. It explores the individual and collective perspectives, attitudes, and experiences of those actively engaged in co-creation efforts. | E.g.:  “I feel the process was fair and open. In my view, it brought people closer together.”  “After I talked with the youth, I realized they felt closer to each other after those sessions”. |
| 16. Experience | This component evaluates the co-creators’ holistic experience within the method or process. It includes, for example, their comfort level, their perception of success, or their enjoyment of the process. | **Use this code whenever the text refers to some general comments about the process and outcomes, like an overview.**  E.g.:  “Overall, it was a positive experience for the team, as we learned a lot.”  “Unfortunately, they were not feeling comfortable and valued during the meetings.” |
| 17. Satisfaction | This component evaluates the level of satisfaction and/or dissatisfaction among the co-creators who participated in the process. | E.g.:  “We could see in their feedback they were really happy with how everything turned out, despite the challenges.”  “I wasn’t fully satisfied with the final product, but I’m glad I took part.” |
| 18. User-friendly execution | This component evaluates the user-friendliness and ease of execution for both the co-creators and the facilitator. It encompasses various considerations, such as the utilization of graphical visualization techniques, metaphors, or plain language, when possible, to enhance co-creators understanding. Additionally, it emphasizes inclusivity by ensuring an intuitive execution that aligns with the diverse needs of all co-creators. | **Use this code whenever the text refers to the protocol aspects that were either challenging or easy to implement, as well as strategies used to apply the original plan.**  **How the co-creation process or methods were experienced in terms of simplicity, ease of use, or smoothness of execution. If the experience of a co-creation activity, method, or tool is being judged as accessible (or not), easy (or not) to follow, intuitive, or confusing, that’s User-Friendly Execution. It can be related to navigating the sessions, activities, the format of the protocol, the cognitive load and efforts needed to make the sessions smooth..**  E.g.:  “The visuals and simple language made it easy to follow.It was straightforward, even for those not used to this kind of work.”  “It was quite tricky to translate all the concepts and abstract models into something tangible for teenagers to grasp.” |
| **CONTEXT** | | |
| 19. Feasibility | This component evaluates the practical aspects and likelihood of success for implementing a co-creation process within a specific setting, considering available resources, potential challenges, and alignment with stakeholders’ needs, perspectives and goals. It encompasses an analysis of factors such as funding, technical infrastructure, and time constraints, alongside identification and mitigation of risks and barriers. | Use this code whenever the text refers to whether the co-creation process or method is realistically doable given the local resources, conditions, or constraints. If someone is talking about whether something is possible, realistic, or workable in the given context, or describing the logistical or practical limitations that affect the implementation — that’s Feasibility.  E.g.:  “The initial proposal was very complex and almost impossible to be executed. After a second round of sessions, we could re-design something much more doable for the current reality.”  “Luckily, we had enough time, money and people to make it happen.”  “We had to run workshops on Saturdays because parents were unavailable during the week”.  “We could not do it during school time.. and it´s very hard for them to participate in this during their free time”. |
| 20. Readiness for change | This component evaluates the degree to which co-creators were prepared to take part in the process and adopt or change their perspectives. For instance, engaging in unfamiliar or abstract methods, or adopting and implementing changes in their behaviour. | E.g.:  “At first, I was hesitant, but I got used to the new ideas quickly.”  “There was no one in the school talking about this when we started the project. We had to open a lot of minds to make people ready to try something different.” |
| 21. Resources and support | This component evaluates the allocation of resources (personnel,funding, technology, etc.), and support mechanisms (training, guidance, etc.), which aim to ensure that the resources align with goals, and support is available and accessible. | **This code has a strong link with code number 12 (Experiential - resource accessibility). Use this one whenever the text is referring to broader contextual resources that were specifically allocated for the intervention.**  E.g.:  “The staff was highly trained in that area even before we started, which really helped.”  “Whenever we needed support, there was always someone from the school to help us out.” |
| 22. Contextual influences | This component evaluates the broader and external social, cultural, economic, and political factors that influence and impact the success or failure of the co-creation process and method in a specific context. It may include consideration of personal influencing factors, such as experiences, personalities, skills and capabilities. | **Use this code when the texts refers to external things existing previously to the intervention and the stakeholders action (social, economic, infrastructure, security, etc.)**  E.g.:  “The local scene really shaped how people responded to this, as they are surrounded with violence everywhere.”  “The challenge was to have the caregivers sign the consent, not because they don't want to, but because, if you consider that most people work such long days, they're just not available to sign a form.”  “The new funding rules made it harder to do everything we planned, as the government significantly cut parts of our funding.” |
| **IMPACT** | | |
| 23. Capacity building | This component evaluates the extent to which a co-creation process developed co-creators’ skills, knowledge, resources, and/or resilience and capabilities.  It also may include the extent to which the process contributed to the capacity of individuals, organisations, or communities to deliver or sustain the implementation of the co-created solution. | E.g.:  “They learned a lot about communication and negotiation skills that can be used in other projects too.”  “Now I feel more confident to take on similar roles in the future.” |
| 24. Maintenance and systems integration | This component evaluates the endurance and assimilation of intervention outcomes and collaborative relationships within the relevant context or system. Maintenance involves the sustained preservation of intervention outcomes and collaborative ties over time. Integration pertains to the incorporation of these outcomes and relationships into the broader system or context addressed by the intervention or co-creation process, whether these are existing structures, practices or policies. | E.g.:  “The changes we made have stuck — they’re part of how we work now.”  “Our new practices have been added into the official policies.”  “Even after the sessions ended, the collaboration between the teacher and the community leader remained strong. They are still in touch whenever there is a need for referrals.” |
| 25. Mechanisms of impact | This component evaluates the underlying factors and processes that drive the specific and potential causal mechanisms or pathways that linked the co- creation process or co-created solution to the observed changes in health-related behaviours, health outcomes, or other targeted outcomes. Within the co-creation process, it explores how co-creation and intersectoral collaboration contribute to these outcomes, while in the implementation of co-created solutions, it investigates the observed changes and the pathways through which they manifest. | **Use this code whenever the text refers to the underlying reasons the intervention/activity had a specific impact.**  E.g.:  “I think a space to be heard is a big thing in this process. That what they say is valued and can go towards creating something. Especially because I think these young people are just not used to being listened to.”  “Bringing people together from different sectors really made a difference for this result, as they could connect in a way they never did.” |
| 26. Knowledge integration and evidence | This component evaluates how well diverse knowledge and evidence are integrated to enhance the co-creation process. It focuses on validating information, balancing expertise, experience, and existing evidence. | E.g.:  “We combined local knowledge with expert advice, which worked well.”  “They made sure to back up ideas with proper data and research.” |
| 27. Empowerment | This component evaluates the extent to which participation in the co- creation process enabled empowerment. This may include effects over the enhancement of participants' agency, autonomy, and self-efficacy; the fostering of equitable decision-making processes and power-sharing mechanisms; the development of leadership skills, capacity-building opportunities, and the creation of supportive environments that enable individuals to exercise control over their circumstances and effect positive change. It may also include the assessment of whether co-creators can become agents of change within their communities or organizations and environment as a result of the co-creation process. | E.g.:  “I feel like I have a say in decisions that affect my neighborhood now.”  “The participants had the sense that they were in charge, to the extent they requested to be part of the execution of the proposal”. |
| 28. Objective alignment | This component evaluates the effectiveness in aligning with the objectives intended to be achieved. It also assesses how well the method or process supports and aligns with the goals and intentions of those involved. The emphasis is on ensuring that the co-creation process and the outcome align harmoniously with the initially intended objectives, contributing to a meaningful alignment between the method or process and the overarching goals. | **This code has a strong link with code number 13 (Experiential - Clarity). Use this one specifically when the text refers to goals and objective and how this alignment helped in the desirable outcomes.**  E.g.:  “Everything we did matched our original goals.”  “The outcome really aligned with what we set out to achieve.” |
| 29. Personal change | This component evaluates shifts in perspectives, enhancement of skills, strengthening of social connections, and other behavioral transformations occurring during the co-creation process; including the employment of specific methods. These changes reflect the individual and collective growth, learning, and development resulting from active engagement in the co-creation process. It also includes self-perceived social, cognitive, and behavioral transformations experienced by co-creators. | E.g.:  “I’ve become more confident speaking in groups and this has increased my positive interactions overall.”  “This experience really changed how they think about community work.” |
| 30. Reach | This component evaluates whether the co-creation process or implementation of co-created solution, successfully reached its intended audience or target co-creators. | E.g.:  “They managed to involve people who normally don’t join in this type of programme.”  “All of these things are strengthening the bonds with the community, whether it's the adolescents, their families or the broader stakeholders.” |
| **DELIVERY** | | |
| 31. Useful outputs | This component evaluates whether the method or process generates outputs that are useful for the co-creators or the process itself. It also evaluates the diverse range of output formats used for visualizing the generated data, acknowledging that different contexts or projects may require distinct formats. | E.g.:  “We produced a toolkit that’s really practical for others to use.”  “The final report is something we can build on in future projects.”  “Because there was flexibility, we could actually come up with something that makes sense for us, so now the proposal can be addressed.”  “If this output is insanely complex, the youth will not engage with it, unfortunately.” |
| 32. Transparency | This component evaluates regarding the inputs, outputs, and procedural steps of a method or process. It evaluates how well it  visibility of its steps, components and how clearly it connects input to outcomes. | E.g.:  “They showed us exactly how decisions were made.”  “I always knew what happened to the data we gave.” |
| 33. Fidelity | This component evaluates the extent to which the completed process or method solution implementation reflects the original aims, designs or protocol; while acknowledging and accommodating potential changes or refinements over time, which occurred in response to new conditions, circumstances or the co- creators feedback. | E.g.:  “It stayed true to the plan, even when things changed.”  “I think it's been a challenge aligning all the projects in different countries. So, even though we had a timeline and protocol, each one had to adjust differently, and this made us a bit insecure.”  “We could not follow the exact session plan because too many changes were needed to fit it into the youth schedule.” |
| 34. Reliability | This component evaluates how well a method or process performs its intended function without failure or deviation. A reliable process consistently produces similar results under similar conditions, which are accurate and in accordance with the intended specifications or requirements. It can also assess the ability to ensure consistency of the solutions, data, or information, providing a basis for decision-making and further analyses or iterations. | E.g.:  “The method worked well each time we used it.”  “We got consistent results whenever we repeated the process.” |
| 35. Impact assessment | This component evaluates the impact of the method or process on the co-creators. For example, it assesses whether co-creators have increased their capacity to engage or acquired additional knowledge. It also evaluates how co- creators influence the process, such as whether the outcomes reflect their contributions. | **This code can be strongly linked to others, like number 23 (Impact - capacity building) and number 29 (Impact - personal change). To reduce overlaps, I suggest avoiding this code or use it only if it’s extremely necessary.**  E.g.:  “They checked with us to see what we learned and how it helped us grow.”  “We could see how our input directly shaped the final outcome.” |
| 36. Efficiency | This component evaluates the overall performance in optimizing time and resources required to produce the desired outcome, while ensuring that the method or process remains impactful and effective in achieving its intended goals. The aim is to streamline the process without compromising effectiveness. | E.g.:  “We got a lot done in a short time without wasting resources.”  “They made good use of everyone’s time and skills.” |
| 37. Adaptation | The process of according to new conditions, circumstances, co-creators feedback, and/or in relation to the original design or protocol. | **Use this code whenever the text refers to elements that had to be adjusted or adapted so the process could flow easier, or elements that suffered modifications from the original plan/idea, for some reason.**  E.g.:  “We changed our approach midway when new connections came up.”  “There was freedom to that, so they adjusted the plan based on what we suggested.” |
